# Hernia

**Published:** 1894-06-23

**Authors:** 


### Progress in Surgery.

### HERNIA.

Mr. Stanmore Bishop draws attention[1] to the necessity of bearing in mind the cause of any given hernia before resorting to operative measures, for even the best-planned operation may end in failure or relapse, if the cause which originally produced the rupture is still in action. To be certain of its previous removal there should be no doubt as to its identity. To assist in determining this the following conclusions are justifiable: (1) That chronic and acute hernia are absolutely different things, agreeing only in the fact that they are both protrusions of viscera through their normal environments; (2) that they differ in etiology, pathology and course; (3) that, especially from the point of view of radical cure, it is important to distinguish between them; (4) that in discussing the feasibility of operations for radical cure, and especially the permanency of their results, the etiology of chronic hernia is of immense importance, since an operation for the cure of the results of a cause is almost certain to be useless whilst the cause remains in operation, and if the cause can be found the patient can be warned to avoid it after the operation has been performed, in order that permanency may be rendered more probable; (5) that in the case of chronic hernia the cause is never one acting suddenly and simply, as overlifting, strains, falls, &c., but always one acting slowly, persistently, gently, habitually (such causes are difficulties in urination and defæcation, certain occupations, and chiefly and most prominently coughing in all its forms; and (6) that the claims of any operation for the radical cure of chronic hernia cannot at present be properly estimated. Mr. Wherry claims[2] a fair amount of success in reducing hernia by manipulation while the patient is coughing, as he believes that the abdominal parts concerned are in the most favourable position for the reduction of hernia while the patient is coughing. Raiford says that 90 per cent. of cases of strangulated hernia can be reduced in a few minutes by the method of treatment which he describes.[3] The operator should be seated on the left of the patient, and should perform taxis while an assistant pours, from a height, cold water over the tumour. With the index and middle fingers he first finds the constriction, the exact localization of which is materially aided by traction made upon the uppermost part of the tumour with the right hand. This should be done simultaneously with the palpation of the stricture, and with the first application of the cold douche. The constriction found, the fingers are gently inserted beneath it, where they are held with a slight pull upwards, while the tumour is lifted upward and pressed inward with the right hand, the cold douche being continually applied. The author has, in his cases, placed the patient in the dorsal position, shoulders lowered, hip elevated, thighs flexed at somewhat more than a right angle with the body. Anæsthesia is unnecessary.

Dr. Morton and Mr. Butler report[4] two rare cases in which the Fallopian tube was included in the contents of the sac in a femoral hernia.

Berger reports[5] two interesting cases of successful operation for the radical cure of congenital umbilical hernia. Both were cases of embryonal omphalocele, and the sac in each case contained the cæcum and the adjacent portion of the ileum; they were partly irreducible because of adhesions between the cæcum and the lining of the sac; both were successfully treated by radical cure—in one, thirty hours, and the other on the fourth day after birth. In cases in which the omphalocele extends into the cord and is due to defective development, dating from the embryonal period, of the abdominal parietes, this author advises immediate operation, the method to be used being a laparotomy, in the course of which the hernia is reduced, adhesions of the contents are broken up, the hernial sac in all its layers is removed, and the abdominal wall united by layers of sutures passing through entirely normal and fully-developed tissues. Chloroform anæsthesia the author believes to be a necessity, and as harmless as in adult life. The only contra-indications to immediate operation are an arrest of development so great that the abdominal wall cannot be brought together by a plastic operation, and the case of children born too long before term or too feeble. The co-existence of other malformations that do not endanger the life of the child is not a contra-indication, but may be taken into account with other considerations.

Bruns, in reporting[6] a case of operation for the radical cure of umbilical hernia, states that the name "omphalectomy" has been given by Keen to a procedure in which the margins of the hernial orifice are cut away. By removing the fibrous tissue of the umbilical ring the surgeon, it is held, can more effectually close this opening and ensure, through direct adhesion of the raw edges, its permanent obliteration. The operation performed by the author is one devised by Condamin as a modification and extension of Keen's method. A curved incision about six inches in length was commenced in the middle line, and carried along the base of the hernia on the right side as far as the corresponding point in the middle line below. In the line of this incision the whole thickness of the abdominal wall, including the peritoneum, was divided. On raising the inner edge of this wound, the inner opening of the hernial sac, through which the tips of two fingers could be passed, was freely exposed. The neck of the sac was now excised, and a short transverse incision made from the middle of the outer edges of the long wound. The sac contained large masses of adherent omentum, which could now be readily detached and returned into the abdominal cavity. The two ends of the curved incision were next joined by a similar curved incision made through the abdominal wall on the left side of the hernia. The sac with its coverings and the soft parts around the hernial ring were then removed. The large wound thus formed was carefully closed by two rows of sutures, one row including the deeper layers of the abdominal wall together with the peritoneum, the other and superficial row bringing together the edges of the skin. Bruns claims for this operation the following advantages: The most difficult stage of an operation for radical cure of hernia—that of returning the contents of the sac—is rendered easier and shorter; the conditions for primary healing of the wound are more favourable, as not only all parts of the hernial tumour, but also the fibrous structures of the ring, which are apt to become gangrenous, are wholly removed; there is less risk of relapse, as the hernial orifice, together with the adjacent linea alba, is completely obliterated.

Rogner-Gussenthal reports[7] a rare case of incarcerated obturator hernia in a patient sixty-six years old, which terminated fatally on the fifth day after operation. At the autopsy it was found that the hernial sac contained the right broad ligament with the tube and ovary, the uterus being drawn over to the obturator foramen. The condition had probably existed for twenty-six years, the history showing that incarceration had occurred on previous occasions. The event showed that a median incision in Trendelenburg's posture would have been preferable to the inguinal operation which was performed. Only three similar cases have been reported.

[1] Lancet, Feb. 10th, 1894. [2] Lancet, Jan. 27th, 1894, p. 204. [3] Americ. Med. Surg. Bulletin and Therapeutic Gazette, Jan., 1894. [4] Lancet. Feb. 17th, 1894, p. 403. [5] Revue de Chir., Oct. 10th, 1893, and Am. Journ. of Med. Sciences, March, 1894. [6] British Med. Journ., Feb. 3rd, 1894. [7] Amer. Journ. Med. Sciences, Nov., 1893, p. 623, and Feb., 1894, p. 211.

